# Galectin-1 and Galectin-9 Concentration in Maternal Serum: Implications in Pregnancies Complicated with Preterm Prelabor Rupture of Membranes

**DOI:** 10.3390/jcm11216330

**Published:** 2022-10-27

**Authors:** Dorota Grażyna Boroń, Aleksy Świetlicki, Michał Potograbski, Grażyna Kurzawińska, Przemysław Wirstlein, Daniel Boroń, Krzysztof Drews, Agnieszka Seremak-Mrozikiewicz

**Affiliations:** 1 Department of Perinatology and Women’s Diseases, Poznań University of Medical Sciences, 60-535 Poznan, Poland; 2 Doctoral School, Poznań University of Medical Sciences, 60-812 Poznan, Poland; 3 Laboratory of Molecular Biology, Department of Perinatology and Women’s Diseases, Poznań University of Medical Sciences, 60-535 Poznan, Poland; 4 Department of Reproduction, Poznań University of Medical Sciences, 60-535 Poznan, Poland

**Keywords:** galectins, pPROM, pregnancy

## Abstract

Preterm prelabor rupture of membranes (pPROM) accounts for nearly half of premature births. Although several risk factors have been identified, no markers allowing for effective prevention have been discovered. In this study, we investigated how the maternal serum levels of galectin-1 and galectin-9 change in patients with pPROM in comparison to uncomplicated pregnancies. A total of 75 patients were enrolled to both study and control group (37 vs. 38, respectively). The serum concentration of galectin-1 and galectin-9 were assayed in duplicate using an enzyme-linked immunoassay. All analyses were performed using PQ Stat v. 1.8.4 software. Galectin-1 levels were significantly higher in the controls (13.32 vs. 14.71 ng/mL, *p* = 0.02). Galectin-9 levels were similar in both groups (13.31 vs. 14.76 ng/mL, *p* = 0.30). Lower galectin levels were detected for early pPROM (before 32nd GW) in comparison to late pPROM and the controls (8.85 vs. 14.45 vs. 14.71 ng/mL, *p* = 0.0004). Similar trend was observed in galectin-9 levels, although no statistical significance was found (11.57 vs. 14.25 vs. 14.76 ng/mL, *p* = 0.26). Low galectin-1 maternal serum level is associated with the incidence of preterm prelabor rupture of membranes. Galectin-9 maternal serum levels were not significantly correlated with pPROM. However, in order to investigate gal-1 and gal-9 levels as potential, promising markers of pPROM, further clinical studies on larger groups are required.

## 1. Introduction

The premature prelabor rupture of fetal membranes (pPROM) is defined as rupture of the membranes before 37 weeks of gestation unrelated to labor [1]. Affecting about 4% of pregnancies worldwide, it accounts for almost half of premature labors (PL)—a global burden, with 1 million children dying before the age of 5 years annually [2,3]. In addition to its high mortality, PL is also associated with prolonged hospital admissions, both in childhood and adult life, and, therefore, with significant costs to health systems [4,5,6,7,8,9,10]. PL is further classified as extremely preterm (<28 gestational weeks (GW)), very preterm (28 to <32 GW) and moderate (32 to <34 GW) to late preterm (34 to <37 GW), with the highest complications and mortality rate in extremely and very preterm neonates [3,11]. Despite great advances in perinatal care in recent decades, the pathophysiological mechanisms leading to pPROM remain uncertain. Several risk factors have been identified, including vaginal infection, multiple pregnancies, polyhydramnios, vaginal bleeding, or smoking [12]. The most widespread view is that pPROM is a complication caused by a preterm activation of the common pathway leading to weakening and eventually to the membranes’ rupture. Microfractures that appear on the surface are portals of entry for pathogens, which result in a possibly life-threatening intrauterine infection [13]. However, we lack specific markers, which may distinguish high-risk groups of pPROM and its most dangerous complications, which could easily be implemented in clinical practice.

Galectins, a group of proteins widely present in mammals, regulate a variety of key biological processes [14]. To date, 13 subtypes have been identified in humans [15]. Their biological function varies depending on the subtype, availability of a suitable ligand and even local concentrations, thus creating functional diversification [16,17]. Expressed in various locations at the materno-fetal site, galectins are mainly involved in immune modulation and early pregnancy events, such as embryo implantation, trophoblast invasion and angiogenesis [18,19]. Therefore, they are responsible for the physiological, healthy course of pregnancy. Due to their immunomodulating function, galectins function as a shield, preventing the rejection of the semi-allogenic fetus and, as a consequence, also enable the termination of the pregnancy [20,21,22]. As previously described in numerous pregnancy complications, including preecalmpsia [23], fetal growth restriction [24] or the premature rupture of membranes [25], the role of galectins remains unclear. The research in this field is still at an early stage and requires further exploration.

In this study, we aimed to investigate maternal serum galectin-1 and galacetin-9 levels in pregnancies complicated with pPROM in comparison to healthy pregnancies delivered at term.

## 2. Materials and Methods

This prospective, single-center study was conducted between June 2020 and May 2022 at the Gynecology and Obstetrics Clinical Hospital in Poznań. A total of 75 women were enrolled, including 37 cases of pPROM (between 23 and 36 weeks of gestation, healthy women with physiological course of the pregnancy beforehand), prior to the administration of any drugs routinely recommended in this clinical situation, and 38 healthy mothers of full-term infants. Cases were enrolled at the hospital’s emergency department during the initial evaluation, after confirmation of pPROM and revision of patient’s general and obstetrical history. Gestational-aged matched pregnant women, admitted to our hospital in the same period, with no obstetric complications or other serious medical conditions, were selected as the control group. Each patient provided a onetime 10 mL sample of the whole blood collected into a K2EDTA tube for further testing. Each sample was centrifuged for 10 min at 2500 rpm and the collected serum was stored at −80 °C until analysis. Exclusion criteria were defined as follows: maternal age of under 18 years, history of drug abuse or cigarette smoking, serious diseases of the mother, including: hypertension, preeclampsia, diabetes, cholestasis of pregnancy, unstable thyroid disease; intrauterine infection, multiple pregnancy, detected fetal or placental abnormalities, fetal growth restriction, cervical insufficiency. Controls were enrolled during a routine check-up of an uncomplicated pregnancy. All subjects were obliged to provide written, informed consent to participate in the study. Medical records of the participants were acquired to obtain more detailed information about the patients.

Gestational age was calculated according to the last menstrual period, or by fetal crown-rump length measurement at the first trimester. pPROM diagnosis was based on the speculum examination (visualization of amniotic fluid in the vagina) or, if necessary, by using a placental insulin growth-factor-binding protein-1 test (Amnioquick, Biosynex Swiss SA). All patients with pPROM were hospitalized and managed according to the following schedule: corticosteroids for lung maturation (if <34 GW) and prophylactic antibiotics were administered, together with a daily cardiotocographic monitoring and fetal assessment. The patients were monitored for any signs of intrauterine infection (CBC and CRP every second day or more frequently, if necessary).

The serum concentration of galectin-1 and galectin-9 were assayed in duplicate using an enzyme-linked immunoassay (ELISA) kit according to the manufacturer’s protocol (Galectin-1 and Galectin-9 Human ELISA Kit, Abcam PLC, Cambridge, UK).

All analyses were performed using PQStat Software 2022 (PQStat v.1.8.4. Poznan, Poland). The Kolmogorov–Smirnov test was used to assess the distribution of all continuous variables. The variables with normal distribution were estimated via means ± standard derivation (SD) and compared with the independent samples Student’s t-test. The Mann–Whitney U-test was used to analyze non-normally distributed variables, and the results were expressed as median and interquartile range. Categorical variables were used through frequency counts and percentage. Correlations between variables were evaluated by the Spearman’s correlation analysis for galecitin-1 and by the Pearson’s correlation analysis for galectin-9. The ANOVA Kruskal–Wallis one-way analysis of variance was used to compare three independent groups for galectin-1 (<32 GW, >32 GW, controls) and ANOVA analysis of variance for galectin-9, respectively. Statistical significance was defined as *p* < 0.05 with a two-tailed test.

## 3. Results

**Figure 1:**
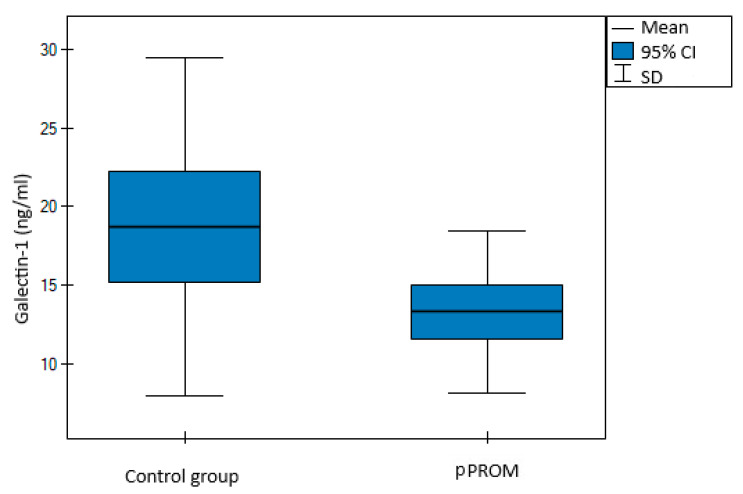
Distribution for maternal serum galectin-1 levels in study vs. control group.

**Figure 2:**
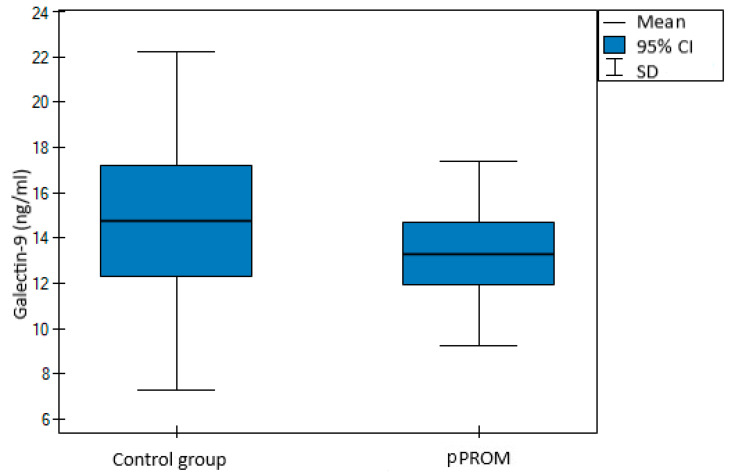
Distribution for maternal serum galectin-9 levels in study vs. control group.

A prospective, case–control study was conducted, involving 75 women, including 37 cases of pPROM and 38 controls. Cases and controls were evenly matched by age. Preterm prelabor rupture of membranes was defined as rupture of membranes between the 23rd and 36th gestational week. The characteristics of the cases and controls are summarized in Table 1. With a similar BMI at blood sampling, C-section rate, female neonate rate and fetal outcomes at birth, defined by pH of the neonate, statistically significant differences were observed with regard to the gestational age at delivery and the birth weight. Surprisingly, although white blood cells (WBC) count and C-reactive protein (CRP) levels were similar, galectin-1 levels were significantly higher in the controls (13.32 vs. 14.71 ng/mL, *p* = 0.02, shown in Figure 1). At the same time, galectin-9 levels were similar in both groups (13.31 vs. 14.76 ng/mL, *p* = 0.30, shown in Figure 2).

**Figure 3:**
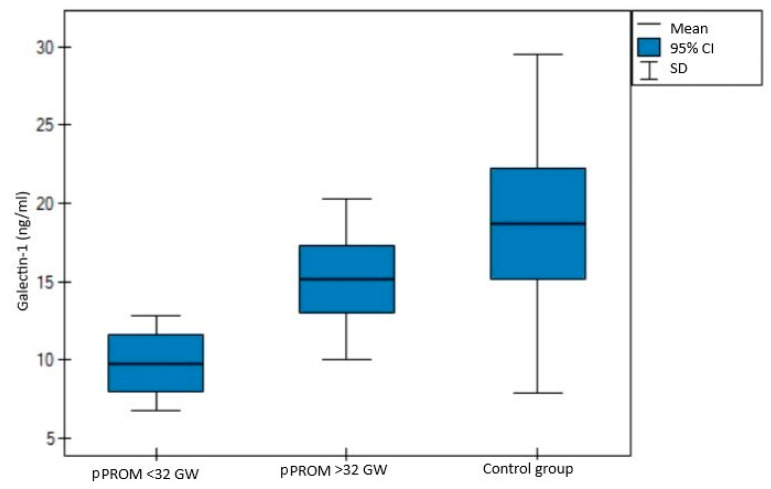
Comparison of three independent groups for galectin-1.

**Figure 4:**
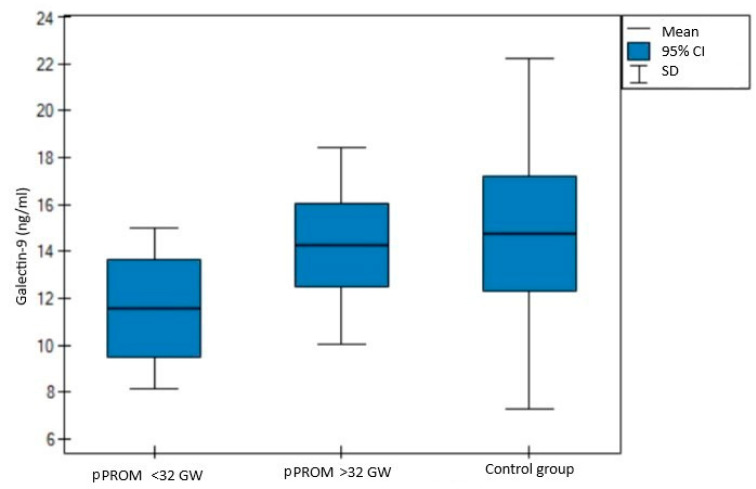
Comparison of three independent groups for galectin-9.

When analyzing the same data, differentiating between early (before 32nd gestational week) and late (after 32nd gestational week) pPROM, the demographic and clinical characteristics were comparable to those described before, indicating even more significant differences in terms of galectin-1 levels. This, in turn, revealed a visible tendency: the earlier the pregnancy is affected by pPROM, the lower the galectin-1 levels (8.85 vs. 14.45 vs. 14.71 ng/mL, *p* = 0.0004, shown in Figure 3). A similar trend was observed in galectin-9 levels, although no statistical significance was observed (11.57 vs. 14.25 vs. 14.76 ng/mL, *p* = 0.26, shown in Figure 4). More detailed data regarding the characteristics with a distinction for early and late pPROM are presented in Table 2.

In order to further investigate the association between pPROM and galectin-1 and 9 levels, the correlations between galectins and other parameters were analyzed (Table 3). Galectin-1 levels were positively correlated with the gestational age at delivery and the birth weight (r = 0.33, *p* = 0.007 and r = 0.32, *p* = 0.008, respectively), whereas galectin-9 levels were significantly related to the BMI of the mother (r = 0.34, *p* = 0.003).

## 4. Discussion

The objective of this study was to investigate the maternal serum levels of galectin-1 and galectin-9 in pregnancies complicated with pPROM. We hypothesized that the maternal serum levels of galectin-1 and galectin-9 would decrease in pregnancies with pPROM, as they both serve as anti-inflammatory molecules, allowing for the maintenance of pregnancy [26,27]. In accordance with our hypothesis, galectin-1 levels were found to be significantly lower in pregnancies with pPROM compared with the control group, especially for early pPROM (<32 GW), which often results in extremely or very preterm labor, which has a huge impact on future care in childhood and adult life. We also found a similar pattern for galectin-9, but with no statistical significance.

Galectin-1 function was widely described in the literature due to its multidirectional effect on all stages of pregnancy. Galectin-1 expression, which is involved in the processes which enable trophoblast attachment to the uterine epithelium [28], is observed in a few-days-old human embryo. Most abundantly expressed in the decidual stromal cells and fetal trophoblasts, gal-1 is sex-hormone-dependent, and thus continuously produced throughout the pregnancy [29]. For instance, galectin-1 is responsible for shaping the phenotype of leukocytes through decidual NK (dNK) cells [30], inducing apoptosis of activated decidual T cells [31] and, thus, ensuring homeostasis at the maternal–fetal interface [15,32]. As maternal serum gal-1 levels significantly increase over the course of pregnancy, it has been suggested as a biomarker for miscarriage, preeclampsia or HELLP syndrome. Nevertheless, none of the previous findings were further implemented in the clinical practice. To the best of our knowledge, only one study investigated the association between serum levels of galectin-1 and pPROM [28] However, Kaya et al., obtained contrasting results, in comparison to ours, demonstrating higher concentrations of gal-1 in patients with pPROM, which is not self-explanatory regardingthe mechanisms of expression of gal-1. Importantly, gal-1 inhibits IL-6 in decidual cells [27], which would account for its anti-inflammatory action, allowing for a favorable microenvironment for maintaining the pregnancy. Additionally, gal-1 expression decreases at term, facilitating the pro-inflammatory changes that lead to the onset of labor [33]. In our study, we hypothesized that maternal serum concentration reflects the expression of gal-1 at the materno-fetal site. Considering the abovementioned reports and our findings, in which we demonstrated that the gal-1 concentration is significantly decreased in patients with pPROM in comparison to the gestational-age-matched controls, this hypothesis may be accurate, suggesting that the presence of galectins in the maternal circulation constitutes a consequence of leakage from the placental tissue. Nevertheless, to fully substantiate this thesis, more detailed studies are essential, involving the placental expression of gal-1 together with serum concentration.

To date, Galectin-9 has been less known, and the data regarding its exact function and characteristics are scarce. Apart from the endometrium, trophoblasts and stromal cells of the decidua [19,34], gal-9 is also expressed by the endothelial cells of the placenta and several types of immune cells [26,35]. Similarly to gal-1, in a murine model gal-9 participates in the processes involved in local anti-inflammatory environment, enabling implantation and early fetal development [34]. Moreover, gal-9 accounts also for the suppression of uterine NK cells by means of secretion in the endometrial stromal cells [26]. Nevertheless, the available data are only based on a murine model, although a similar role for gal-9 has been proposed in view of human pregnancy. As the pregnancy progresses, both the expression and concentration levels of gal-9 in the maternal blood increases, rendering it another possibly crucial galectin in the maintenance of pregnancy [36]. In terms of the available data concerning pregnancy complications, several studies have suggested the role of low gal-9 expression and/or maternal serum levels in spontaneous abortions [26], recurrent pregnancy loss [37,38] and preeclampsia [39,40]. Interestingly, some studies have indicated that levels of gal-9 serum level is higher in women carrying a male fetus, as compared to a female fetus [41,42]. Furthermore, as far as pPROM is concerned, gal-9 expression in the chorion is reduced at the site of the membrane weakening, resulting in rupture. In contrast, to our knowledge, the serum levels of gal-9 in pPROM have not been investigated to date. Despite their having a similar hypothesis as gal-1, we did not confirm a significant difference in gal-9 serum levels in maternal serum in pPROM patients as compared to the healthy controls, even when differentiated into the early and late preterm membrane rupture.

This study has several limitations, which mainly include the small number of participants and single-center character. Additionally, the examination of placenta and fetal membranes may constitute an additional asset in the future.

To conclude, low galectin-1 maternal serum level is associated with the incidence of preterm prelabor rupture of membranes (especially before the 32nd gestational week), either as a trigger factor or one of the molecules on the cascade of events leading to fetal membranes’ damage. Conversely, galectin-9 maternal serum levels are most probably not significantly associated with incidence of pPROM. However, to investigate gal-1 and gal-9 levels as potential, promising clinical markers for the prediction of pPROM, further clinical studies on larger groups are required.

## Figures and Tables

**Table 1:** Demographic and clinical characteristics of the study population.

	pPROM (n = 37)	Control (n = 38)	*p* Value
Age (years)	31.62 ± 5.12	28.95 ± 6.26	0.05
Gravida *	2	2	0.11
BMI at blood sampling (kg/m^2^)	26.13 ± 4.04	26.56 ± 4.626	0.67
GA at blood sampling	32.30 ± 3.08	30.29 ± 3.27	**0.008**
GA at delivery	34.43 ± 2.72	38.47 ± 1.14	**<0.00001**
C-section (%) **	35.14	26.67	0.46
Female neonate (%) **	35.14	50	0.22
Birth weight (g)	2407.97 ± 575.35	3456.50 ± 383.94	**<0.000001**
pH of the neonate at birth	7.27 ± 0.08	7.27 ± 0.06	0.65
WBC count	12.59 ± 3.11	10.81 ± 2.35	0.08
CRP	5.76 ± 6.80	5.91± 2.97	0.80
Galectin-1 (ng/mL) *	13.32 (6.29)	14.71 (10.34)	**0.02**
Galectin-9 (ng/mL)	13.307 ± 4.09	14.76 ± 7.47	0.30

pPROM—preterm prelabour rupture of membranes; GA—gestational age; BMI—body mass index; WBC—white blood cells; CRP—C-reactive protein. * Non-normally distributed values shown as median (interquartile range). ** Categorical variables shown as percentage. Bold font indicates statistical significance.

**Table 2:** Demographic and clinical characteristics with distinction for early (<32 GW) and late (>32 GW) pPROM.

	pPROM < 32 (n = 13)	pPROM > 32 (n = 24)	Control (n = 38)	*p* Value
Age (years)	31.69 ± 3.33	31.58 ± 5.94	28.95 ± 6.26	0.14
Gravida *	2	2	2	0.11
BMI at blood sampling (kg/m2)	25.73 ± 4.29	26.34 ± 3.98	26.56 ± 4.63	0.84
GA at blood sampling	28.85 ± 2.44	34.17 ± 1.17	30.29 ± 3.27	**<0.000001**
GA at delivery	33.08 ± 3.68	35.17 ± 1.71	38.47 ± 1.14	**<0.000001**
C-section (%) **	38.46	31.53	26.67	0.15
Female neonate (%) **	30.77	38.12	50	0.45
Birth weight (g)	2185.38 ± 754.98	2528.54 ± 421.39	3456.50 ± 383.94	**<0.000001**
pH of the neonate at birth	7.26 ± 0.07	7.8 ± 0.06	7.27 ± 0.06	0.84
WBC count	13.34 ± 3.24	12.18 ± 3.03	10.81 ± 2.35	0.11
CRP	7.19 ± 6.87	4.98 ± 6.78	5.19 ± 2.97	0.57
Galectin-1 (ng/mL) *	8.85 (3.54)	14.45 (5.14)	14.71 (10.34)	**0.0004**
Galectin-9 (ng/mL)	11.57 ± 3.42	14.25 ± 4.18	14.76 ± 7.47	0.26

GW—gestational week; pPROM—preterm prelabour rupture of membranes; GA—gestational age; BMI—body mass index; WBC—white blood cells; CRP—C-reactive protein. * Non-normally distributed values shown as median (interquartile range). ** Categorical variables shown as percentage. Bold font indicates statistical significance.

**Table 3:** Correlactions between galectins levels and other parameters. Spearman’s correlation analysis was used for galectin-1 and Pearson’s correlation analysis for galectin-9.

	Galectin-1	Galectin-9
Age	R = 0.18	R = −0.03
	*p* = 0.12	*p* = 0.80
BMI	R = 0.11	R = 0.34
	*p* = 0.33	*p* = **0.003**
GA at blood sampling	R = 0.15	R = 0.08
	*p* = 0.19	*p* = 0.52
GA at delivery	R = 0.33	R = 0.17
	***p* = **0.007****	*p* = 0.15
Birth weight	R = 0.32	R = 0.09
	***p* = **0.008****	*p* = 0.49
pH of the neonate at birth	R = −0.11	R = 0.14
	*p* = 0.38	*p* = 0.25
WBC	R = −0.25	R = 0.04
	*p* = 0.09	*p* = 0.79
CRP	R = 0.33	R = 0.04
	*p* = 0.82	*p* = 0.79

GA—gestational age; BMI—body mass index; WBC—white blood cells; CRP—C-reactive protein. Bold font indicates statistical significance.

## Data Availability

All data generated or analyzed during this study are included in this article. Further enquiries can be directed to the corresponding author.
